# Prevalence of high-risk group for obstructive sleep apnea using the STOP-Bang questionnaire and its association with cardiovascular morbidity

**DOI:** 10.3389/fneur.2024.1394345

**Published:** 2024-12-09

**Authors:** Jieun Kang, Hyeon-Kyoung Koo, Hyung Koo Kang, Woo Jung Seo, Jiyeon Kang, Jinseob Kim

**Affiliations:** ^1^Division of Pulmonary and Critical Care Medicine, Department of Internal Medicine, Ilsan Paik Hospital, Inje University School of Medicine, Goyang, Republic of Korea; ^2^Zarathu Co., Ltd., Seoul, Republic of Korea

**Keywords:** obstructive sleep apnea, STOP-Bang questionnaire, risk assessment, morbidity, cardiovascular disease

## Abstract

**Objectives:**

Obstructive sleep apnea (OSA) is a common sleep-related breathing disorder, yet many cases remain undiagnosed. The STOP-Bang questionnaire was developed to identify individuals at high risk of OSA. We aimed to investigate the prevalence of individuals with suspected OSA using the STOP-Bang risk stratification in the general population of South Korea. Additionally, we determined if the STOP-Bang risk stratification independently predicts cardiovascular morbidity.

**Methods:**

Data from the eighth Korea National Health and Nutrition Examination Survey (2019–2020) were used. Participants aged ≥40 with complete data for STOP-Bang questionnaire were included. A STOP-Bang score of ≥5 classified individuals as high-risk whereas scores of 3–4 and less than 3 classified them as intermediate- and low-risk, respectively. The association between the high-risk group and cardiovascular morbidity was analyzed using complex sample logistic regression.

**Results:**

Among the 6,630 participants included, approximately 6.7% were classified as high-risk based on the STOP-Bang questionnaire. The prevalence of diagnosed OSA in the high-risk group was 4.0%. The high-risk group showed a significantly higher prevalence of cardiovascular morbidity (11.9%) compared to those in the low- and intermediate-risk groups (3.0 and 8.1%, respectively). After adjusting for variables associated with cardiovascular risk, the high-risk group remained an independent predictor of increased likelihood of cardiovascular morbidity compared to the low-risk group (odds ratio, 2.05; *p* = 0.002). When stratified by sex, STOP-Bang high-risk was significantly associated with cardiovascular morbidity in men; however, the same trend was not observed in women.

**Conclusion:**

We found a significant proportion of individuals at high risk of OSA is likely to remain undiagnosed in the general population of South Korea. The high-risk group demonstrated a higher burden of cardiovascular morbidity, and the STOP-Bang high-risk group was an independent predictor of cardiovascular morbidity.

## 1 Introduction

Obstructive sleep apnea (OSA) is a highly-prevalent sleep disorder characterized by repeated episodes of apnea or hypopnea caused by upper airway obstruction during sleep (1). According to a recent literature-based analysis, the prevalence of OSA, defined by apnea-hypopnea index (AHI) ≥ 5, was estimated to exceed 50% in some countries, although variations exist by country and region (2). Previous studies that utilized in-laboratory polysomnography reported that the prevalence of OSA in the general population ranges from 13 to 33% in men and 6 to 19% in women, with higher rates in advanced age groups (3). Similarly, in South Korea, the prevalence of OSA has been reported to be 27 and 17% in men and women aged 40–69 years, respectively (4). However, owing to the nature of apnea and hypopnea events that occur during sleep, many patients with OSA may be unaware of their condition and remain undiagnosed (5). It has been estimated that more than 80% of individuals with moderate-to-severe OSA remain undiagnosed (6).

OSA is strongly associated with an increased risk of traffic accidents, hypertension, stroke, coronary artery disease, and mortality (7–10). In managing OSA, early identification and intervention are crucial to prevent such adverse consequences. Polysomnography, the gold-standard diagnostic method, is expensive and requires specialized equipment and personnel, rendering it less accessible to many individuals. Even if a polysomnography is ordered, there are long waiting times in most cases (11). To identify individuals at high risk of OSA, several screening tools have been developed (12–15). Of them, the snoring, tiredness, observed apnea, hypertension, body mass index (BMI), age, neck circumference, and gender (STOP-Bang) questionnaire has demonstrated high sensitivity in detecting moderate-to-severe OSA (15). The sensitivity and specificity of the STOP-Bang questionnaire in detecting OSA with an apnea–hypopnea index ≥15 were 92.9 and 43.0%, respectively (15). Furthermore, higher STOP-Bang scores indicate a higher probability of OSA (16, 17).

In a previous study, Braley et al. found that 56% of Americans aged 65 years or older were estimated to be at high risk of OSA, using a sleep questionnaire similar to the STOP-Bang questionnaire (18). Despite the significant prevalence of the high-risk group, only 8% underwent OSA testing, with 95% receiving confirmation of OSA. The STOP-Bang questionnaire can help identify individuals at high risk of OSA and potentially predict cardiovascular morbidity. However, the potential of STOP-Bang risk stratification in predicting cardiovascular morbidity has received limited attention. This study aimed to investigate the prevalence of individuals classified as high-risk in the general population of South Korea using the STOP-Bang questionnaire, comparing it to the prevalence of confirmed OSA. Furthermore, it also evaluated STOP-Bang risk stratification as an independent predictor of cardiovascular morbidity.

## 2 Materials and methods

### 2.1 Data source and participants

The Korea National Health and Nutrition Examination Survey (KNHANES) is a nationwide population-based cross-sectional survey conducted annually by the Korea Centers for Disease Control and Prevention to assess the health and nutritional status of the Korean population. The KNHANES, designed as a complex sample survey using a multistage sampling method to represent the general, non-institutionalized Korean population, includes health interviews, health examinations, and nutritional surveys. The detailed study protocol has been previously described (19). This study used a dataset from the eighth (2019–2020) KNHANES. In the eighth KNHANES, of the 20,808 individuals selected for the survey, 15,469 completed it, yielding a response rate of 74.3%.

In the health interview, the STOP questionnaire was completed by participants aged ≥40 years. Participants with complete STOP-Bang questionnaire data were included in our analysis. Written informed consent to participate in the study was obtained from all participants. This study was approved by the Institutional Review Board of the Ilsan Paik Hospital (No. 2022-09-032).

### 2.2 STOP-Bang questionnaire

The STOP-Bang questionnaire comprised four questions from the STOP questionnaire, along with four additional demographic questions, resulting in eight dichotomous questions (yes = 1 point; no = 0 point) (15). The four questions in the STOP questionnaire are on snoring, tiredness, observed apnea, and high blood pressure: (1) Do you snore loudly? (2) Do you often feel tired, fatigued, or sleepy during the daytime? (3) Has anyone observed you stop breathing or choking/gasping during your sleep? (4) Do you have high blood pressure? The remaining four demographic and anthropometric characteristics include BMI > 35 kg/m^2^, age > 50 years, neck circumference > 40 cm, and male sex. The total score is calculated by summing the scores of all eight questions, ranging from 0 to 8. Higher scores indicate a higher risk of OSA: scores of 0–2, 3–4, and 5–8 indicate a low, intermediate, and high risk, respectively (i.e., conventional scoring system) (5).

The STOP questionnaire and different versions of the STOP-Bang questionnaire were also employed in this study. The STOP questionnaire classifies individuals into a high-risk group when two of the four questions are positive (15). Compared to the conventional STOP-Bang scoring system, an alternative scoring model has been demonstrated to enhance the specificity of OSA detection (20). Using the alternative scoring model, individuals with an intermediate risk of OSA (STOP-Bang score of 3–4) are classified as a high-risk group if they meet any of the following three combinations: (1) a STOP score ≥ 2 and a BMI >35 kg/m^2^, (2) a STOP score ≥ 2 and a neck circumference > 40 cm, and (3) a STOP score ≥ 2 and male sex. Furthermore, modified cut-off for BMI (>30 kg/m^2^) and neck circumference (>36.3 cm) have been suggested as useful measures for screening of OSA in the Korean population (modified STOP-Bang for Koreans) (21). These alternative risk stratification methods as well as scores of the STOP-Bang questionnaire were investigated for their association with cardiovascular morbidity.

### 2.3 Data collection

Data obtained included demographic characteristics and socioeconomic status (educational level, household income level, and type of medical insurance). Participants’ social history data, including information on smoking status, alcohol consumption habits, and level of physical activity, were obtained through interviews. Household income levels were classified into quartiles. Regular physical activity was defined as engagement in moderate-intensity physical activity for ≥2 h and 30 min per week, engagement in high-intensity physical activity for ≥1 h and 15 min per week, or a combination of moderate- and high-intensity activities (where 1 min of high-intensity activity was equivalent to 2 min of moderate-intensity activity) (22). In addition, existing comorbidities or medical conditions were documented. Anthropometric measurements, such as blood pressure, height, weight, and neck and waist circumferences, were recorded.

### 2.4 Outcomes

The study aimed to investigate the prevalence of individuals at high risk of OSA based on the STOP-Bang questionnaire in the general population of South Korea. The objective was to assess the proportion of potential OSA patients without a confirmed diagnosis. Furthermore, this study sought to evaluate the predictive value of the STOP-Bang risk stratification for cardiovascular morbidity. Cardiovascular morbidity was defined as the composite prevalence of stroke, myocardial infarction, and angina. The likelihood of cardiovascular morbidity was further analyzed by stratification according to sex and the use of different OSA risk prediction methods, including the STOP questionnaire and different versions of the STOP-Bang questionnaire.

### 2.5 Statistical analysis

As the KNHANES dataset was produced using a complex, multistage, stratified, probability sampling, all statistical analyses were performed using complex sample analyses with stratification variables, clustering variables, and weighted values. Categorical variables were compared using the Rao-Scott corrections to the Pearson chi-squared test and presented as weighted percentages. Continuous variables were compared using the Complex survey design *t*-test and presented as weighted numbers with standard errors. Complex sample logistic regression analysis was used to calculate odds ratios (OR) with 95% confidence intervals (CI) for cardiovascular morbidity. To explore whether the STOP-Bang high-risk group independently predicts cardiovascular morbidity, other variables that are considered to affect cardiovascular morbidity were adjusted in the multivariable model. Multivariable analyses were conducted adjusting for variables that were statistically significant in unadjusted analyses. The goodness-of-fit of the regression models were evaluated using the Akaike information criterion (AIC). Given that the STOP-Bang questionnaire incorporates sex, sex-stratified analysis was performed. In addition to the conventional STOP-Bang questionnaire, we employed the STOP questionnaire (15), an alternative scoring model (20), and a modified version of the STOP-Bang for the Korean population (21) to assess the risk of cardiovascular morbidity. All tests were two-tailed, and statistical significance was set at *p* < 0.05. All statistical analyses were performed using the R software (version 4.4.0, R Foundation for Statistical Computing, Vienna, Austria).

## 3 Results

### 3.1 Prevalence of high-risk of OSA among study participants

**Figure 1:**
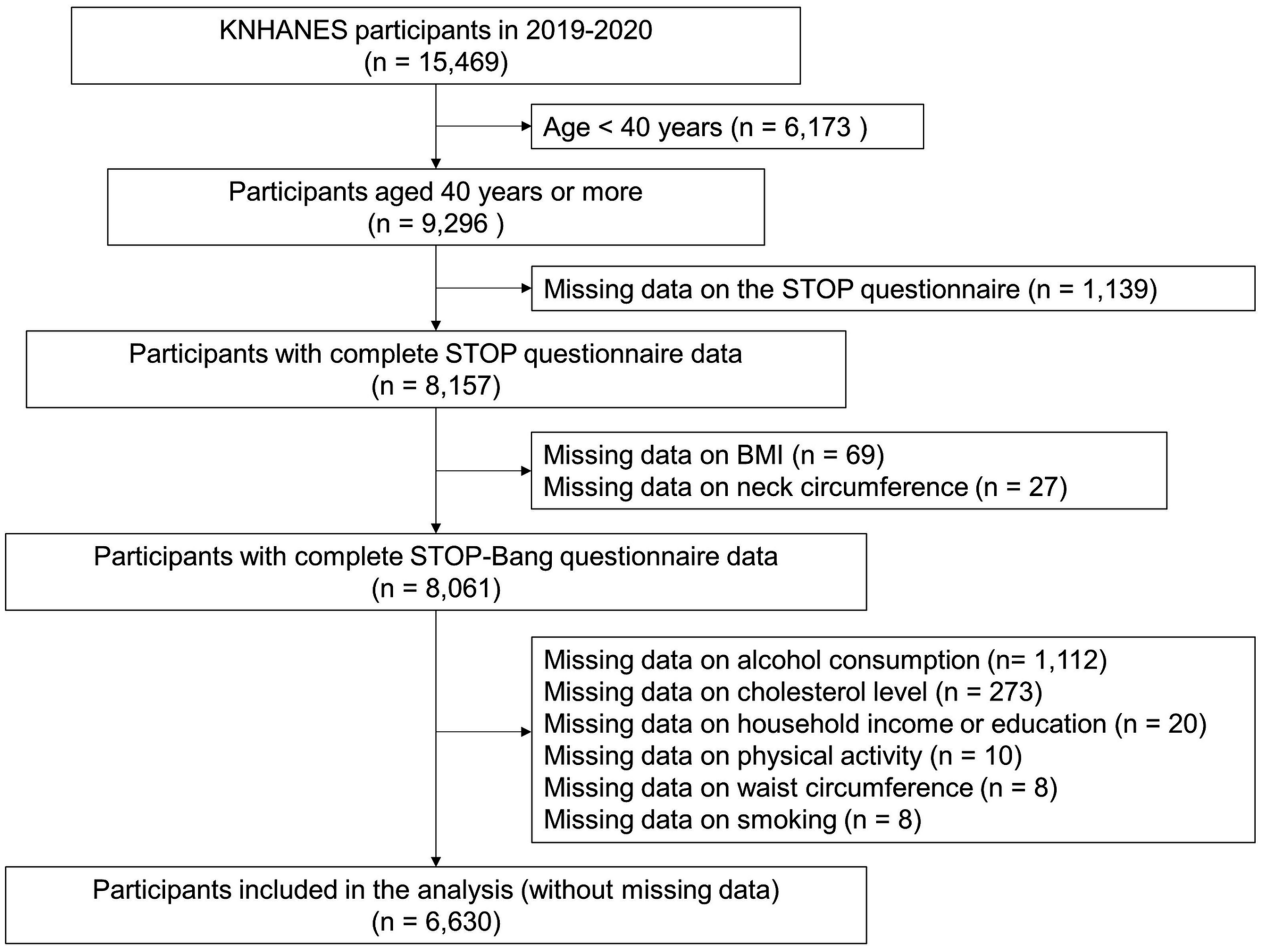
Study participants. KNHANES, Korea National Health and Nutrition Examination Survey; STOP, snoring, tiredness, observed apnea, and hypertension; BMI, body mass index; STOP-Bang, snoring, tiredness, observed apnea, hypertension, body mass index, age, neck circumference, and gender.

A total of 6,630 participants with complete STOP-Bang questionnaire data were included in the analysis (Figure 1). Supplementary Table S1 shows the distribution of the STOP-Bang scores among the study participants. The largest number of patients had a score of 2 (28.5%), followed by 3 (21.9%) and 1 (21.7%). An estimated 6.7% of the total participants were classified into the STOP-Bang high-risk group (a STOP-Bang score ≥ 5).

Among men, 12.4% were classified into the high-risk group based on the STOP-Bang questionnaire whereas 38.0 and 49.6% were classified into the low- and intermediate-risk groups, respectively (Supplementary Table S2). Among women, 83.9, 15.5, and 0.5% were classified into the low-, intermediate-, and high-risk groups, respectively.

**Figure 2:**
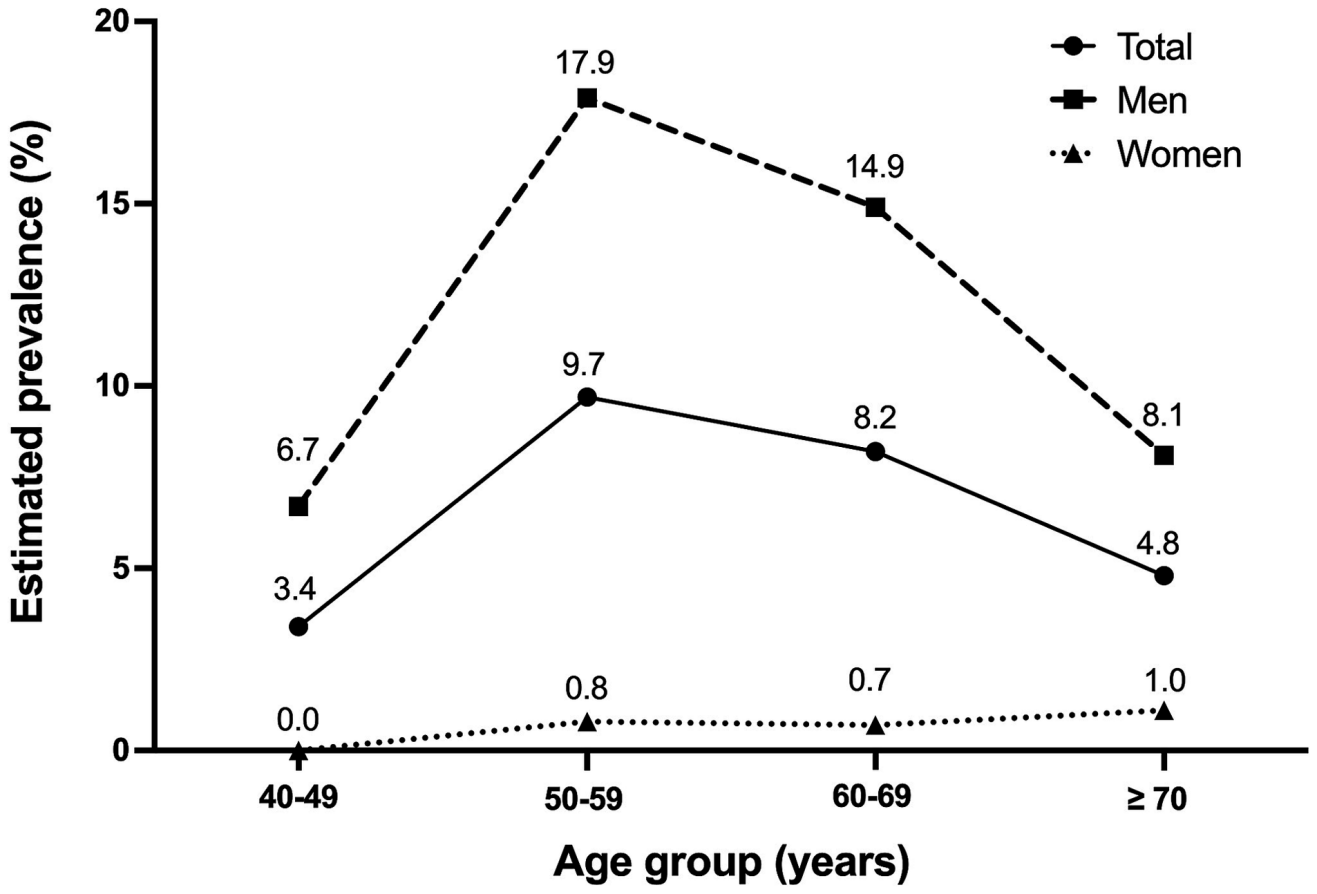
Prevalence of STOP-Bang high-risk group among different age groups. The distribution of STOP-Bang high-risk group prevalence among participants of different age groups is presented for the total population, as well as for men and women. STOP-Bang, snoring, tiredness, observed apnea, hypertension, body mass index, age, neck circumference, and gender.

The proportion of individuals in the STOP-Bang high-risk group peaked among those in their 50s and decreased with advancing age (Figure 2). The proportion of men in the high-risk group was higher than that of women in all age groups. For men, the highest prevalence was observed among those in their 50s, and its prevalence decreased with advancing age. However, among women, there was a trend of an increasing proportion in the high-risk group with age.

### 3.2 Clinical characteristics of high-risk group vs. low- and intermediate-risk groups

The baseline demographic and clinical characteristics of the STOP-Bang low-, intermediate-, and high-risk groups are shown in Table 1. Due to the nature of the STOP-Bang questionnaire, the high-risk group included more men and showed higher mean BMI and neck circumference than the other groups. It also had a higher proportion of current and former smokers and frequent alcohol drinkers (≥2 drinks per week). Additionally, individuals with higher educational levels were more prevalent in the high-risk group.

**Table 1:** Characteristics of study participants based on the STOP-Bang questionnaire grouping.

	Low risk	Intermediate risk	High risk	*p*-value
Prevalence (%)	3,949	2,258	423	
Age (years)	53.6 ± 10.2	59.6 ± 10.5	57.3 ± 8.5	<0.001
Age group				<0.001
40–49	1,480 (43.4)	288 (17.3)	56 (16.9)	
50–59	1,084 (29.8)	628 (35.7)	162 (47.8)	
60–69	853 (17.6)	694 (27.6)	131 (26.3)	
≥70	532 (9.2)	648 (19.4)	74 (9.1)	
Sex (%)				<0.001
Male	1,088 (32.9)	1,660 (77.6)	404 (96.3)	
Female	2,861 (67.1)	598 (22.4)	19 (3.7)	
BMI, kg/m^2^	23.5 ± 3.0	25.1 ± 3.3	27.2 ± 3.5	<0.001
Obesity (BMI ≥25) (%)	1,131 (28.4)	1,053 (48.3)	301 (73.3)	<0.001
Neck circumference, cm	34.2 ± 2.9	37.3 ± 2.9	40.0 ± 2.6	<0.001
Waist circ, cm	82.4 ± 8.8	89.4 ± 8.6	95.8 ± 9.0	<0.001
Systolic blood pressure	116.6 ± 14.9	126.7 ± 15.9	127.2 ± 14.7	<0.001
Diastolic blood pressure	75.6 ± 8.8	79.5 ± 10.3	82.1 ± 11.3	<0.001
Diabetes (%)	330 (7.4)	405 (16.0)	105 (22.2)	<0.001
Dyslipidemia (%)	1,081 (25.6)	827 (35.7)	169 (41.6)	<0.001
Smoking (%)				<0.001
Current	483 (14.2)	505 (24.9)	128 (32.3)	
Former	691 (18.9)	935 (41.7)	233 (54.2)	
Never	2,775 (66.9)	818 (33.4)	62 (13.5)	
Alcohol consumption (%)				<0.001
None–1 drink/month	2,448 (59.7)	1,036 (43.4)	148 (33.5)	
2–4 drinks/month	817 (21.0)	490 (23.5)	91 (22.2)	
2–3 drinks/week	519 (14.6)	460 (21.1)	110 (28.0)	
≥4 drinks/week	165 (4.6)	272 (12.0)	74 (16.3)	
Regular physical activity (%)	1,589 (41.1)	885 (40.2)	185 (41.8)	0.780
Household income (%)				<0.001
Low	607 (12.5)	525 (18.5)	75 (13.7)	
Middle low	942 (22.7)	598 (24.8)	100 (22.5)	
Middle high	1,122 (30.3)	563 (26.9)	109 (26.1)	
High	1,278 (34.5)	572 (29.8)	139 (37.7)	
Education (%)				<0.001
Elementary school	655 (12.3)	580 (19.5)	61 (8.9)	
Middle school	428 (9.1)	338 (13.7)	59 (11.8)	
High school	1,423 (38.6)	701 (32.7)	147 (38.0)	
College or graduate school	1,443 (40.0)	639 (34.1)	156 (41.2)	
Insurance type (%)				<0.001
Medicaid	109 (2.3)	114 (4.0)	24 (4.6)	
Health insurance	3,840 (97.7)	2,144 (96.0)	399 (95.4)	

Data are presented as unweighted number (weighted %) or weighted mean ± standard error.

STOP-Bang, snoring, tiredness, observed apnea, hypertension, body mass index, age, neck circumference, and gender; BMI, body mass index.

### 3.3 Prevalence of confirmed OSA and comorbidities

**Figure 3:**
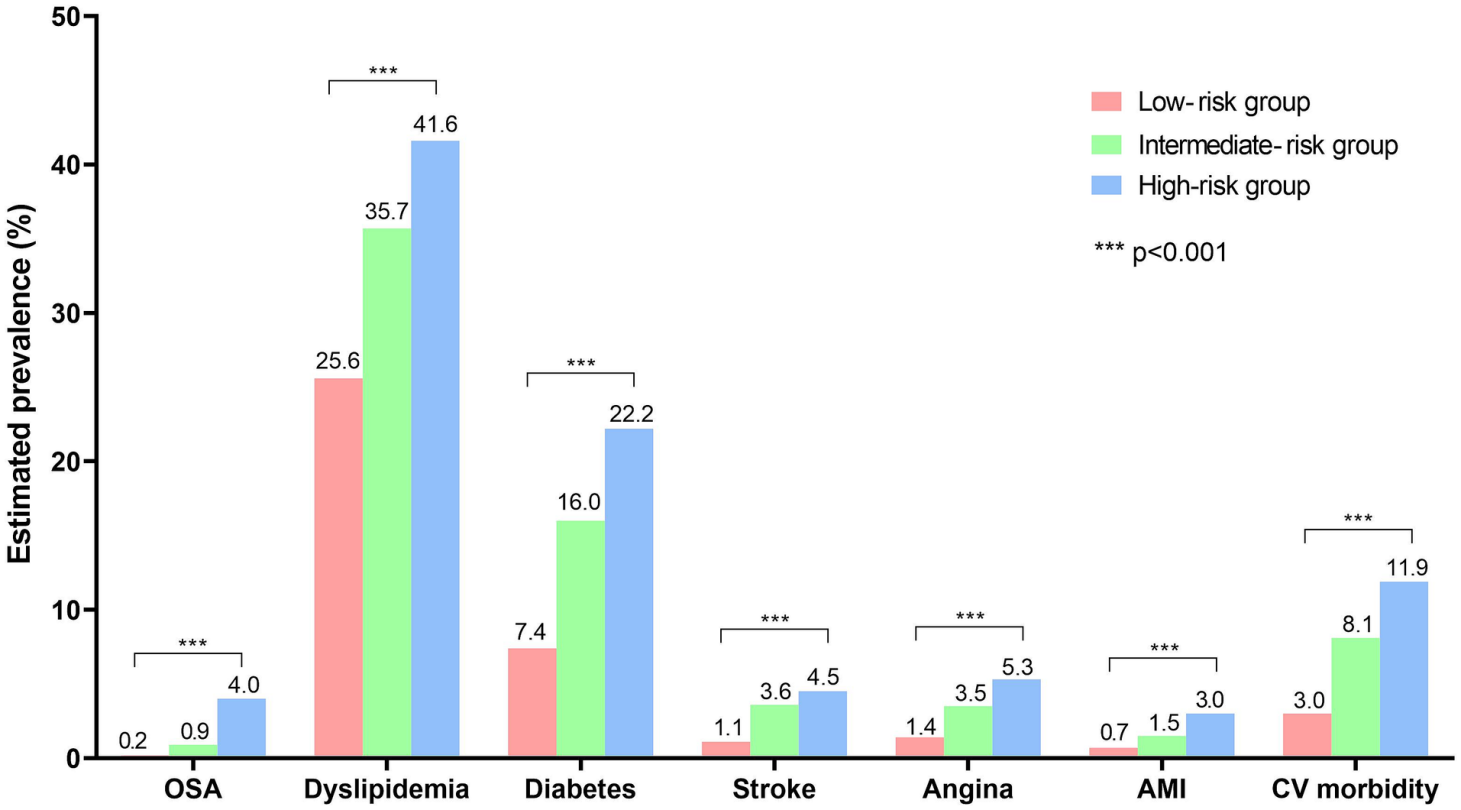
Prevalence of confirmed OSA and associated comorbidities in low-, intermediate-, and high-risk groups. The prevalence of diagnosed obstructive sleep apnea (OSA) and other comorbidities is shown. The prevalence was compared using the Rao-Scott corrections to the Pearson chi-squared test, and all comorbidities shown in the figure exhibited significant differences between the three groups (*p* < 0.001). OSA, obstructive sleep apnea; AMI, acute myocardial infarction; CV, cardiovascular.

The prevalence of confirmed OSA and associated comorbidities is compared in Figure 3. Only 0.6% of participants reported being diagnosed with OSA. The prevalence of diagnosed OSA was 0.2, 0.9, and 4.0% in the low-risk, intermediate-risk, and high-risk groups, respectively. Comorbidities, such as dyslipidemia, diabetes, stroke, angina, and acute myocardial infarction, were significantly more frequent in the high-risk group than in the other two groups. The prevalence of cardiovascular morbidity increased progressively from the low-risk (3.0%) to the intermediate-risk (8.1%) and high-risk groups (11.9%), with a significant difference between the groups (*p* < 0.001).

### 3.4 Association of STOP-Bang risk stratification with cardiovascular morbidity

Table 2 shows the factors associated with cardiovascular morbidity in our study participants. In the unadjusted analysis, older age, male sex, higher BMI, ever smoking, lack of regular physical activity, diabetes, and dyslipidemia were significant risk factors for cardiovascular morbidity. Additionally, individuals in the intermediate- or high-risk groups, compared to the low-risk group, showed a significant association with cardiovascular morbidity. After adjusting for significant variables from the unadjusted analysis, the STOP-Bang high-risk group showed a significantly increased likelihood of cardiovascular morbidity compared to the low-risk group (adjusted OR, 2.05; 95% CI, 1.29–3.24; *p* = 0.002).

**Table 2:** Risk of cardiovascular morbidity among all study participants.

	Unadjusted model			Adjusted model		
	Crude OR	95% CI	*p*-value^a^	Adjusted OR	95% CI	*p*-value^b^
Age	1.08	1.07–1.10	<0.001	**1.08**	**1.06–1.09**	**<0.001**
Sex, male	2.22	1.75–2.83	<0.001	1.51	0.99–2.32	0.056
BMI	1.06	1.02–1.09	0.001	1.03	0.99–1.07	0.185
Ever smoker	1.97	1.55–2.51	<0.001	1.39	0.95–2.03	0.090
Alcohol drinking	1.00	0.76–1.32	0.992			
Regular physical activity	0.77	0.61–0.98	0.034	0.87	0.67–1.12	0.286
Diabetes	3.17	2.45–4.09	<0.001	**1.67**	**1.26–2.22**	**<0.001**
Dyslipidemia	1.87	1.47–2.37	<0.001	**1.42**	**1.11–1.81**	**0.006**
STOP-Bang group						
Low-risk	1.00 (reference)			1.00 (reference)		
Intermediate-risk	2.82	2.18–3.65	<0.001	1.30	0.94–1.79	0.107
High-risk	4.32	3.02–6.20	<0.001	**2.05**	**1.29–3.24**	**0.002**
AIC	2645.77			2406.26		

a Crude *p*-value.

b Adjusted *p*-value.

OR, odds ratio; CI, confidence interval; STOP-Bang, snoring, tiredness, observed apnea, hypertension, body mass index, age, neck circumference, and gender; BMI, body mass index; AIC, Akaike information criterion. The bold texts indicate statistically significant values.

### 3.5 STOP-Bang risk stratification and cardiovascular morbidity stratified by sex

In men, the unadjusted analysis showed that STOP-Bang risk stratification was significantly linked to cardiovascular morbidity, alongside the older age, diabetes, and dyslipidemia (Table 3). The STOP-Bang risk grouping remained as a significant predictor for cardiovascular morbidity after adjusting for age, diabetes, and dyslipidemia; the high-risk group showed a significantly higher risk of cardiovascular morbidity compared to the low-risk group (adjusted OR 2.22; 95% CI, 1.41–3.50; *p* < 0.001).

**Table 3:** Risk of cardiovascular morbidity in men.

	Unadjusted model			Adjusted model		
	Crude OR	95% CI	*p*-value^a^	Adjusted OR	95% CI	*p*-value^b^
Age	1.07	1.06–1.09	<0.001	**1.07**	**1.05–1.09**	**<0.001**
BMI	1.02	0.97–1.07	0.420			
Ever smoker	1.44	0.93–2.25	0.106			
Alcohol drinking	0.76	0.55–1.04	0.087			
Regular physical activity	0.75	0.55–1.02	0.066			
Diabetes	2.59	1.88–3.57	<0.001	**1.59**	**1.13–2.23**	**0.008**
Dyslipidemia	1.81	1.31–2.50	<0.001	**1.49**	**1.09–2.05**	**0.014**
STOP-Bang group						
Low-risk	1.00 (reference)					
Intermediate-risk	2.25	1.53–3.33	<0.001	1.34	0.90–1.99	0.151
High-risk	3.27	2.09–5.12	<0.001	**2.22**	**1.41–3.50**	**<0.001**
AIC	1585.84			1466.57		

a Crude *p*-value.

b Adjusted *p*-value.

OR, odds ratio; CI, confidence interval; STOP-Bang, snoring, tiredness, observed apnea, hypertension, body mass index, age, neck circumference, and gender; BMI, body mass index; AIC, Akaike information criterion. The bold texts indicate statistically significant values.

Among women, the unadjusted analysis showed that the STOP-Bang high- and intermediate-risk groups had significantly higher risk of cardiovascular morbidity compared to the low-risk group. However, STOP-Bang risk stratification did not demonstrate significant association with cardiovascular morbidity after adjusting for age, BMI, diabetes and dyslipidemia (Table 4).

**Table 4:** Risk of cardiovascular morbidity in women.

	Unadjusted model			Adjusted model		
	Crude OR	95% CI	*p*-value^a^	Adjusted OR	95% CI	*p*-value^b^
Age	1.10	1.08–1.12	<0.001	**1.00**	**1.07–1.12**	**<0.001**
BMI	1.08	1.04–1.13	<0.001	1.03	0.97–1.09	0.272
Ever smoker	0.92	0.53–1.60	0.766			
Alcohol drinking	0.80	0.38–1.67	0.547			
Regular physical activity	0.75	0.51–1.11	0.154			
Diabetes	4.20	2.54–6.94	<0.001	**1.88**	**1.03–3.44**	**0.041**
Dyslipidemia	2.39	1.66–3.44	<0.001	1.28	0.85–1.94	0.233
STOP-Bang group						
Low-risk	1.00 (reference)					
Intermediate-risk	2.81	1.81–4.36	<0.001	1.32	0.84–2.07	0.230
High-risk	6.66	1.73–25.67	0.006	2.68	0.47–15.23	0.267
AIC	993.74			884.52		

a Crude *p*-value.

b Adjusted *p*-value.

OR, odds ratio; CI, confidence interval; STOP-Bang, snoring, tiredness, observed apnea, hypertension, body mass index, age, neck circumference, and gender; BMI, body mass index; AIC, Akaike information criterion. The bold texts indicate statistically significant values.

### 3.6 Alternative screening tools and their relationship to cardiovascular morbidity

The STOP questionnaire, alternative STOP-Bang scoring model, and modified STOP-Bang for Koreans identified 25.5, 17.0, and 13.5% of the participants at high risk of OSA, respectively (Supplementary Table S2). Using the STOP questionnaire, the likelihood of cardiovascular morbidity was 1.4-fold higher in the high-risk group compared to the low-risk group (Table 5). Using the alternative STOP-Bang scoring model and modified STOP-Bang for Koreans, the high-risk group exhibited an approximately 1.7-fold and 1.9-fold higher likelihood of cardiovascular morbidity compared to the low-risk group, respectively. Each additional increase in the STOP-Bang score corresponded to a 1.2-fold higher likelihood of cardiovascular morbidity.

**Table 5:** Risk of cardiovascular morbidity of the high-risk group classified by different screening methods.

	Total			Men			Women		
	Adjusted OR^a^	95% CI	*p*-value	Adjusted OR^b^	95% CI	*p*-value	Adjusted OR^c^	95% CI	*p*-value
STOP questionnaire									
Low-risk	1.00 (reference)			1.00 (reference)			1.00 (reference)		
High-risk	1.45	1.12–1.88	0.006	1.53	1.14–2.05	0.005	1.39	0.89–2.18	0.144
AIC	2405.64			1468.14			882.02		
Alternative STOP-Bang scoring^d^									
Low-risk	1.00 (reference)			1.00 (reference)					
Intermediate-risk	1.25	0.90–1.72	0.181	1.27	0.84–1.92	0.257	1.33	0.85–2.09	0.215
High-risk	1.70	1.14–2.52	0.009	1.76	1.18–2.63	0.006	2.18	0.41–11.65	0.361
AIC	2407.80			1468.97			884.75		
Modified STOP-Bang for Koreans^e^									
Low-risk	1.00 (reference)			1.00 (reference)			1.00 (reference)		
Intermediate-risk	1.43	0.96–2.13	0.075	1.84	0.95–3.56	0.069	1.24	0.77–1.99	0.385
High-risk	1.92	1.17–3.16	0.010	2.50	1.28–4.87	0.007	2.07	0.60–7.14	0.247
AIC	2408.45			1467.91			884.83		
STOP-Bang score^f^	1.20	1.06–1.35	0.003	1.21	1.06–1.37	0.004	1.28	1.05–1.55	0.014
AIC	2403.81			1466.64			878.86		

a Adjusted for age, sex, body mass index, ever smoking, regular physical activity, and presence of diabetes and dyslipidemia.

b Adjusted for age and presence of diabetes and dyslipidemia.

c Adjusted for age, body mass index, and presence of diabetes and dyslipidemia.

d Individuals with an intermediate risk of OSA (STOP-Bang score of 3–4) are classified as high-risk if they meet any of the following three combinations: (1) a STOP score ≥ 2 and a BMI > 35 kg/m^2^, (2) a STOP score ≥ 2 and a neck circumference > 40 cm, and (3) a STOP score ≥ 2 and male sex.

e Modified cut-off for BMI (>30 kg/m^2^) and neck circumference (>36.3 cm) are used.

f Indicates a risk for each increase in the STOP-Bang score.

OR, odds ratio; CI, confidence interval; STOP, snoring, tiredness, observed apnea, hypertension; STOP-Bang, snoring, tiredness, observed apnea, hypertension, body mass index, age, neck circumference, and gender; OSA, obstructive sleep apnea; BMI, body mass index; AIC, Akaike information criterion.

When stratified by sex, in men, being classified as the high-risk group using the STOP questionnaire, alternative scoring model, or modified STOP-Bang for Koreans was significantly associated with an increased risk of cardiovascular morbidity (Table 5). In women, each additional point on the STOP-Bang score was significantly associated with an increase in the likelihood of cardiovascular morbidity. However, risk stratification using the STOP questionnaire, alternative scoring model, or modified STOP-Bang for Koreans did not show significant association with cardiovascular morbidity.

## 4 Discussion

In this study, we found that approximately 6.7% of South Korean adults aged ≥40 years were identified as being at high risk of OSA based on the STOP-Bang questionnaire. Surprisingly, only 0.6% of the total participants reported being diagnosed with OSA. Even among those in the high-risk group, only 4.0% were reportedly diagnosed with OSA, indicating a significant proportion of potentially undiagnosed cases within the high-risk group. This high-risk group exhibited a significantly higher cardiovascular morbidity than the low- and intermediate-risk group. After adjusting for other variables that could influence the cardiovascular risk, the STOP-Bang high-risk group demonstrated a two-fold higher likelihood of cardiovascular morbidity than the low-risk group. When stratified by sex, among men, the high-risk group showed a 2.2-fold higher risk of having cardiovascular morbidity than the low-risk group. These findings suggest that being classified as high-risk according to the STOP-Bang questionnaire could independently predict a greater burden of cardiovascular morbidity.

### 4.1 Underdiagnosis of OSA

Using population-based data, our study revealed that only a small proportion of individuals in the STOP-Bang high-risk group had an established diagnosis of OSA, indicating a substantial level of potential underdiagnosis. It is estimated that more than 80% of individuals with OSA remain undiagnosed in the community (6). According to a report by Frost and Sullivan, commissioned by the American Academy of Sleep Medicine, approximately 5.9 million adults have been diagnosed with OSA whereas 23.5 million remain undiagnosed in the United States (22). Although geographic variations may exist in the proportion of undiagnosed patients, it is important to note that economic and healthcare burdens are significant for patients who remain undiagnosed or untreated for OSA, even in South Korea (22–24). Therefore, efforts are needed to reduce the gap between diagnosed and undiagnosed patients in the high-risk group, including improving awareness of the disease in the general population.

### 4.2 Age-related prevalence of STOP-Bang high-risk group

While the highest prevalence was observed among men in their 50s, a consistent rise in high-risk prevalence with age was noted among women. This pattern may be linked to the elevated prevalence of OSA in women after menopause. Several cohort studies have consistently shown a higher OSA prevalence in postmenopausal women compared to premenopausal women. In the Wisconsin Sleep Cohort Study, Young et al. demonstrated a 3.5-fold increased risk for moderate-to-severe OSA in postmenopausal women compared to premenopausal women (25). Similarly, Mirer et al. revealed a 31% higher apnea–hypopnea index in postmenopausal women compared premenopausal women (26). This observation may be attributed to postmenopausal weight gain, resulting in increased BMI and neck circumference, leading to anatomical changes in the upper airway and compromised breathing during sleep. However, increased body weight alone may not fully explain the heightened risk of OSA. An alternative mechanism suggests that reductions in estrogen and/or progesterone could induce instability in the ventilatory control system, resulting in an overall increased risk of OSA (27).

### 4.3 STOP-Bang risk stratification as a predictor for cardiovascular morbidity

As OSA is well-recognized as an independent risk factor for cardiovascular diseases (28), early diagnosis and treatment are important. Utilizing the STOP-Bang questionnaire may help identify patients with OSA who should receive prioritized interventions to prevent adverse cardiovascular outcomes, even before polysomnography confirmation. Parameters like age, obesity, and gender, incorporated into the STOP-Bang questionnaire, have been linked to cardiovascular outcomes. While some studies have suggested an association between self-reported snoring and an increased risk of cardiovascular disease (29, 30), conflicting findings have surfaced in other research (31, 32). However, it remains unclear whether the STOP-Bang questionnaire can accurately estimate cardiovascular morbidity related to OSA. In a prior study by Niiranen et al., a set of questions on sleep-disordered breathing was employed to classify patients with self-reported OSA in Finnish adults aged 30 or older (33). This study revealed that self-reported OSA was an independent predictor of cardiovascular events. However, simple self-reported snoring alone did not exhibit any association with future cardiovascular events. Another study examined the correlation of the STOP-Bang questionnaire with major cardiovascular events in a hospitalized population (34). This investigation suggested that although the high-risk group, defined by a STOP-Bang score of 5 or more, experienced a higher incidence of major adverse cardiovascular events compared to the low-risk group, this relationship lost statistical significance after adjusting for other confounding factors in the multivariate model. Our study differs from prior research in several ways. We used the STOP-Bang questionnaire and focused on the general population rather than hospitalized patients, with a notably larger sample size. Our findings indicated that the high-risk group was two times more likely to have cardiovascular morbidity compared to the low-risk group, even after accounting for other risk factors. This underscores the importance of active screening and appropriate treatment in high-risk individuals. Given the advantages of the STOP-Bang questionnaire, which is simpler to administer than the costly and labor-intensive polysomnography, its wider use should be considered.

In contrast to the results in men, the STOP-Bang risk grouping did not exhibit a significant association with cardiovascular morbidity in our female population. Since the STOP-Bang questionnaire takes gender into account, it is more likely to categorize men as high-risk. Consequently, the number of females classified as high-risk in our study was very small, with only 0.5% of the total female participants falling into this category. While it is possible that the STOP-Bang risk grouping may not effectively predict cardiovascular morbidity in women, the limited number of females classified as high-risk may have contributed to the lack of statistical power in this regard. Further analyses in different populations are necessary to address this concern.

The utilization of the STOP questionnaire or alternative versions of the STOP-Bang resulted in a higher proportion of individuals being classified as high-risk compared to the conventional STOP-Bang questionnaire. An alternative scoring model in which the intermediate-risk group meeting the criteria of BMI >35 kg/m^2^, neck circumference > 40 cm, or being male with a STOP score ≥ 2 is classified as high-risk has been shown to have a lower sensitivity but increased specificity compared to the conventional STOP-Bang (20). However, it has been suggested that the BMI >35 kg/m^2^ and neck circumference > 40 cm criteria used in the STOP-Bang may be unsuitable for Koreans. Because the STOP-Bang was developed and validated in Western countries, its direct application in Asian populations can be challenging. Asians tend to be less obese than Caucasians, even in cases of severe OSA (35, 36). In this regard, a modified version using BMI >30 kg/m^2^ and neck circumference > 36.3 cm as the criteria has been proposed for Korean populations (21). According to the findings of our study, a significantly higher likelihood of cardiovascular morbidity was observed in the high-risk group compared to the low-risk group, regardless of the method used. The AIC values were generally similar across the various scoring systems assessed. Therefore, irrespective of the type of questionnaire, if an individual is classified as high-risk (or has a high STOP-Bang score), appropriate testing and intervention for OSA should be pursued due to its significant association with cardiovascular morbidity.

### 4.4 Limitations

Our study has several limitations that need to be addressed. First, although we aimed to address the gap between suspected (STOP-Bang high-risk group) and confirmed OSA patients, not all individuals in the high-risk group necessarily had OSA confirmed using polysomnography. Therefore, the extent of the gap may have been overestimated. Nevertheless, considering that only 0.6% of all participants in the KNHANES cohort, which is considered representative of the Korean population, reported an OSA diagnosis, it is undeniable that OSA remains significantly underdiagnosed in South Korea. Second, our analysis was limited to prevalent cardiovascular diseases, because our study design was cross-sectional population-based analysis. Analyses of incident cardiovascular diseases or risk of future cardiovascular events data were not possible. The significance of our study lies in the fact that individuals in the high-risk group were likely to have a greater burden of cardiovascular disease. Although a strong association between OSA and adverse cardiovascular outcomes has been widely recognized, further research is warranted to determine whether the STOP-Bang alone can predict future cardiovascular outcomes independently. Third, our analysis did not include adults younger than 40 years because the questionnaires assessing snoring, tiredness, and observed apnea were administered only to participants aged 40 and older. While OSA can occur in younger individuals, cardiovascular morbidity increases with age. Therefore, we believe that our analysis was conducted on a relevant population. Fourth, it is important to note that the STOP-Bang questionnaire incorporates factors, such as age, hypertension, and obesity, which can potentially influence cardiovascular risk. Despite our efforts to adjust for these confounding factors, the possibility of unaccounted for confounding effects remains. Finally, our analysis was conducted only on Korean participants, making it challenging to extrapolate our findings to other ethnic groups. The validation of our findings in cohorts representing different ethnicities is necessary to ascertain the generalizability of our results.

## 5 Conclusion

In conclusion, our study demonstrated that a significant proportion of individuals at high risk of OSA could remain undiagnosed in the general population of South Korea. The high-risk group showed a higher burden of cardiovascular morbidity, and the STOP-Bang high-risk group was an independent predictor of cardiovascular morbidity. Efforts should be made to reduce the gap between diagnosed and undiagnosed patients in the high-risk group, and active screening using the STOP-Bang questionnaire may be helpful in the general population.

## Data Availability

Publicly available datasets were analyzed in this study. This data can be found here: http://knhanes.cdc.go.kr.
