# Supplementary material for: Prevalence of high-risk group for obstructive sleep apnea using the STOP-Bang questionnaire and its association with cardiovascular morbidity
Source: Front Neurol. 2024 Dec 9;15:1394345. doi: 10.3389/fneur.2024.1394345 (PMC11663855; doi:10.3389/fneur.2024.1394345)
Supplement: Supplementary file 1 [file Table_1.DOCX]

Supplementary Material

Prevalence of High-Risk Group for Obstructive Sleep Apnea Using the STOP-Bang Questionnaire and its Association with Cardiovascular Morbidity

Jieun Kang*, Hyeon-Kyoung Koo, Hyung Koo Kang, Woo Jung Seo, Jiyeon Kang

*** Correspondence:** Jieun Kang: [realodette@gmail.com](mailto:realodette@gmail.com)

# Supplementary Table 1. Distribution of STOP-Bang scores in the study population

| STOP-Bang score | Number | Percent |
| --- | --- | --- |
| 0 | 610 | 9.8 |
| 1 | 1404 | 21.7 |
| 2 | 1935 | 28.5 |
| 3 | 1525 | 21.9 |
| 4 | 733 | 11.3 |
| 5 | 289 | 4.5 |
| 6 | 109 | 1.8 |
| 7 | 24 | 0.4 |
| 8 | 1 | 0.0 |

Abbreviation: STOP-Bang, snoring, tiredness, observed apnea, hypertension, body mass index, age, neck circumference, and gender.

# Supplementary Table 2. Risk classification according to the STOP-Bang, STOP, alternative scoring model, and modified version of the STOP-Bang for Koreans

|  | Total | Men | Women | P-value |
| --- | --- | --- | --- | --- |
| STOP-Bang questionnaire |  |  |  | <0.001 |
| Low-risk | 3949 (60.1) | 1088 (38.0) | 2861 (83.9) |  |
| Intermediate-risk | 2258 (33.3) | 1660 (49.6) | 598 (15.5) |  |
| High-risk | 423 (6.7) | 404 (12.4) | 19 (0.5) |  |
| STOP questionnaire​ |  |  |  | <0.001 |
| Low-risk | 4947 (74.5) | 2152 (67.9) | 2795 (81.7) |  |
| High-risk | 1683 (25.5) | 1000 (32.1) | 683 (18.3) |  |
| Alternative STOP-Bang scoring^​a^ |  |  |  | <0.001 |
| Low-risk | 3949 (60.1) | 1088 (38.0) | 2861 (83.9) |  |
| Intermediate-risk | 1657 (23.0) | 1064 (29.9) | 593 (15.4) |  |
| High-risk | 1024 (17.0) | 1000 (32.1) | 24 (0.6) |  |
| Modified STOP-Bang for Koreans^b^ |  |  |  | <0.001 |
| Low-risk | 3258 (48.3) | 505 (18.0) | 2753 (81.1) |  |
| Intermediate-risk | 2523 (38.3) | 1858 (57.7) | 665 (17.2) |  |
| High-risk | 849 (13.5) | 789 (24.3) | 60 (1.7) |  |

^a^Individuals with an intermediate risk of OSA (STOP-Bang score of 3–4) are classified as high-risk if they meet any of the following three combinations: (1) a STOP score ≥2 and a BMI >35 kg/m^2^, (2) a STOP score ≥2 and a neck circumference >40 cm, and (3) a STOP score ≥2 and male sex.

^b^Modified cut-off for BMI (>30 kg/m^2^) and neck circumference (>36.3 cm) are used.

Abbreviation: STOP-Bang, snoring, tiredness, observed apnea, hypertension, body mass index, age, neck circumference, and gender; STOP, snoring, tiredness, observed apnea, hypertension; OSA, obstructive sleep apnea; BMI, body mass index.
